# Editorial: Current Progress and Challenges in the Development of a B Cell Based Hepatitis C Virus Vaccine

**DOI:** 10.3389/fimmu.2018.02577

**Published:** 2018-11-09

**Authors:** Steven K. H. Foung, Thomas F. Baumert

**Affiliations:** ^1^Department of Pathology, Stanford University School of Medicine, Stanford, CA, United States; ^2^Inserm, U1110, Institut de Recherche sur les Maladies Virales et Hépatiques, Strasbourg, France; ^3^Université de Strasbourg, Strasbourg, France; ^4^Institut Hospitalo-Universitaire, Pôle Hépato-digestif, Hôpitaux Universitaires de Strasbourg, Strasbourg, France; ^5^Institut Universitaire de France, Paris, France

**Keywords:** hepatitis (C) virus, virus neutralization, vaccine, monoclonal abs, epitopes, humoral immunity

More than 70 million people worldwide are infected with hepatitis C virus (HCV), a major cause of liver cirrhosis, liver failure and hepatocellular carcinoma (HCC) world-wide. In the last decade, HCC has emerged as the second leading cause of cancer death. The World Health Organization estimates an increase in the global burden by two million new infections per year and mainly due to injection drug use (IDU). Infection is increasing in young adults in the U.S. because of IDU (1). For patients, the development of highly efficient HCV-specific direct acting antivirals (DAAs) has markedly improved treatment and disease outcome. However, the high costs of DAA limit their access to patients with low income or limited health insurance and in countries with limited resources (2). Indeed these challenges are reasons why overall access to DAA has been estimated to be <10% of the HCV-infected patients on a global level (3). Moreover, the absent access of the majority of patients translates into very limited effect on the global disease burden such as HCV-induced HCC (4). In addition, health care workers with occupational risk for blood-borne pathogens and injection drug users (IDUs) will remain at risk for repeated exposure to HCV, even after successful treatment. This is dramatically illustrated by the growing number of HCV infections in the opioid epidemic (5). Recent clinical evidence suggests that treatment-induced cure in patients in advanced fibrosis does not eliminate the risk of HCC [for review see (6)]. These challenges strongly suggest that DAAs will neither be sufficient to eradicate the disease on a global level nor in distinct patient populations such as IDUs. Taken together, there is a significant need for an effective preventive HCV vaccine to be developed. The articles in this research topic describe the progress that has been made toward a preventive vaccine and the challenges that still need to be overcome to ultimately achieve this goal.

A first step in a “rational vaccine design” approach for HCV is to identify relevant mechanisms of immune correlates of protection. Naglaa Shoukry from the University of Montreal summarizes the challenges to vaccine development and the efforts required to overcome them. Multiple lines of evidence suggest that CD4+ and CD8+ T cell responses are needed to control acute infection but are insufficient for preventing long-term persistence. At the same time, cumulative evidence supports the importance of virus neutralizing antibodies to protect against HCV infection and to facilitate clearance. Most anti-HCV antibodies are directed mainly against the E2 glycoprotein and some to E1 and E1E2. Both envelope proteins are required for viral entry. The function of E2 has been studied in great detail, however much less is known about its “partner in crime,” envelope glycoprotein E1 and the interactions of E1 and E2. While Tong et al. from the Institut Pasteur of Shanghai describe the function of E1 and aspects of its structure and function that are important for HCV vaccine design, Yost et al. from the University of New Jersey and from the NIH present the complexities of the flexible E2 and E1E2 heterodimer glycoproteins and how the flexibility of disordered regions on the glycoproteins might affect vaccine development. In that regard, computer models of the HCV glycoproteins (E1 and E2) that describe the interplay of E1 and E2 and potential interactions of E1/E2 with host HCV receptors could inform about new approaches to rational vaccine design (reviewed by Guest and Pierce from the University of Maryland and by Kinchen and Bailey from the John Hopkins University School of Medicine).

A significant challenge for a B cell based HCV vaccine is defining conserved epitopes that are capable of eliciting protective antibodies unassociated with viral escape. Keck et al. from Stanford University give a comprehensive overview of the immunogenicity of E2 and summarize epitopes that could be targeted for rational vaccine design. The hypervariable region 1 in the HCV E2 glycoprotein, HVR1, is an immunodominant region associated with neutralization and viral escape as reviewed by Prentoe and Bukh from the University of Copenhagen. Substantial efforts have shown that the majority of antibodies with broad neutralizing activities to diverse HCV isolates recognize conformational epitopes in the HCV E2 glycoprotein, as reviewed by Tzarum et al. from The Scripps Research Institute. Of particular importance is the region of the CD81 binding domain. Ströh et al. from Hanover Medical School focus on the flexibility of this neutralization defining region and discuss the impact for vaccine design. Only some of these conserved epitopes are not associated with viral escape. Cowton et al. from the University of Glasgow identified viral epitopes that were conserved among all strains, giving a promising perspective toward the development of a broadly effective HCV vaccine. An important determinant for viral escape are N-linked glycans. Lavie et al. from the University of Lille highlight the role of N-linked glycans for HCV neutralization and viral escape and give an outlook how modifications of specific glycosylation sites could improve the immunogenicity of vaccine candidates. New strategies will also have keep in mind the close association of HCV with components of the lipid metabolism. In that regard, Wrensch et al. from the University of Strasbourg discuss the interactions of the HCV particle with apolipoproteins and discuss their impact on HCV vaccine design. Vaccine development also requires standardized and sensitive methods two assess the efficacy of vaccine lead candidates. A specific challenge, the development of appropriate test system, that reflects the whole variety of HCV envelope variants is addressed by Kinchen and Bailey from the Johns Hopkins University School of Medicine who stress the need to use extensive well-characterized HCVcc or HCVpp to define neutralization potency and breadth of B cell responses. Furthermore, *in vivo* testing is a key requirement for vaccine development. Burm et al. from Ghent University review existing animal systems for HCV vaccine research and discuss the suitability of liver xenograft models as well as HCV homologs to test vaccine candidates and to assess humoral and cellular immune responses.

In summary, the articles published within this research topic not only give a highly comprehensive overview of the challenges of viral immune evasion required to address for vaccine design, but also informs on the current stage of HCV vaccine research highlighting perspectives and opportunities for the future.

## Author contributions

All authors listed have made a substantial, direct and intellectual contribution to the work, and approved it for publication.

### Conflict of interest statement

The authors declare that the research was conducted in the absence of any commercial or financial relationships that could be construed as a potential conflict of interest.
